# Supplementary material for: Assembly of CNS Nodes of Ranvier in Myelinated Nerves Is Promoted by the Axon Cytoskeleton
Source: Curr Biol. 2017 Apr 3;27(7):1068–73. doi: 10.1016/j.cub.2017.01.025 (PMC5387178; doi:10.1016/j.cub.2017.01.025)
Supplement: Document S1. Figure S1 [file mmc1.pdf]

Current Biology, Volume *27*

Supplemental Information

Assembly of CNS Nodes of Ranvier in Myelinated Nerves Is Promoted by the Axon Cytoskeleton

Veronica Brivio, Catherine Faivre-Sarrailh, Elior Peles, Diane L. Sherman, and Peter J. Brophy

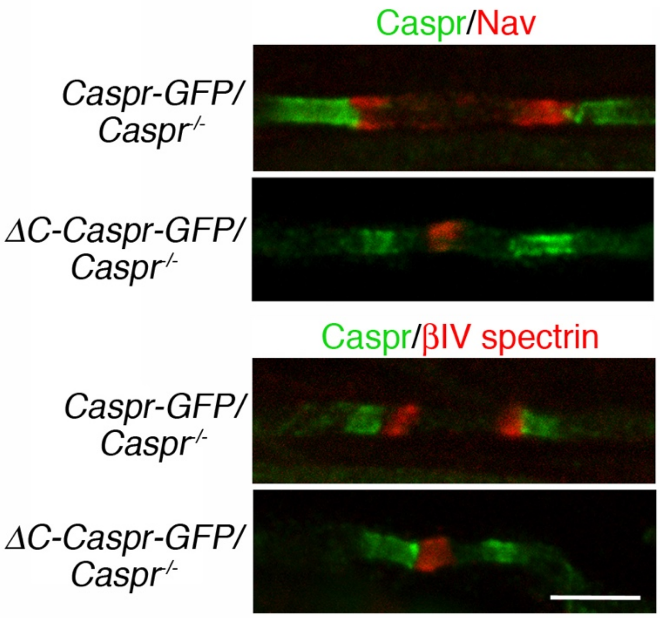

Figure S1. The absence of the Protein 4.1B binding domain of Caspr also impairs the formation of heminodal clusters in the PNS. Related to Figure 2.
Immunofluorescence analysis at P3 of sciatic nerves from *Caspr-GFP/Caspr*$^{-/-}$ and *ΔC-Caspr-GFP/Caspr*$^{-/-}$ mice for Caspr and the nodal proteins Nav and βIV spectrin. Quantitation of $\geq$ 63 pairs of converging Schwann cells for the nodal proteins Nav, βIV spectrin or ankyrinG for each genotype showed that the percentages of symmetrical heminodes were as follows *Caspr-GFP/Caspr*$^{-/-}$, 89.0 ± 0.3 (n = 2); *C-Caspr-GFP/Caspr*$^{-/-}$, 12.0 ± 3.0 (n = 3); mean ± SEM, P $\leq$ 0.0002, Unpaired Student's t test. The scale bar represents 5 μm.
