# Supplementary material for: Assembly of CNS Nodes of Ranvier in Myelinated Nerves Is Promoted by the Axon Cytoskeleton
Source: Curr Biol. 2017 Apr 3;27(7):1068–73. doi: 10.1016/j.cub.2017.01.025 (PMC5387178; doi:10.1016/j.cub.2017.01.025)
Supplement: Document S2. Article plus Supplemental Information [file mmc2.pdf]

Report

Current Biology

Assembly of CNS Nodes of Ranvier in Myelinated Nerves Is Promoted by the Axon Cytoskeleton

Highlights

- Nodes of Ranvier are essential axonal domains in the vertebrate nervous system
- An axoglial adhesion complex promotes node formation by oligodendroglia in the CNS
- The axoglial adhesion complex is anchored to the axon cytoskeleton
- Loss of this anchorage disrupts the axon cytoskeleton and delays node formatfion

Authors

Veronica Brivio,
Catherine Faivre-Sarrailh, Elior Peles,
Diane L. Sherman, Peter J. Brophy

Correspondence

peter.brophy@ed.ac.uk

In Brief

Brivio et al. show that an axoglial adhesion complex drives domain formation at nodes of Ranvier by organizing the axon cytoskeleton at the boundary between converging glial processes and nodal complexes at heminodes. Heminodes fuse to form the mature node, thus ensuring efficient formation of nodes of Ranvier and rapid nerve impulse conduction.

CrossMark

Brivio et al., 2017, Current Biology *27*, 1068–1073
April 3, 2017 © 2017 The Author(s). Published by Elsevier Ltd.
http://dx.doi.org/10.1016/j.cub.2017.01.025

CellPress

Current Biology
Report

Assembly of CNS Nodes of Ranvier in Myelinated Nerves Is Promoted by the Axon Cytoskeleton

Veronica Brivio,[1] Catherine Faivre-Sarrailh,[2] Elior Peles,[3] Diane L. Sherman,[1] and Peter J. Brophy[1,4,*]
[1]Centre for Neuroregeneration, University of Edinburgh, Edinburgh EH16 4SB, UK
[2]Centre de Recherche en Neurobiologie et Neurophysiologie de Marseille-UMR 7286, CNRS, 13344 Marseille, France
[3]Department of Molecular Cell Biology, The Weizmann Institute of Science, Rehovot 76100, Israel
[4]Lead Contact
*Correspondence: peter.brophy@ed.ac.uk
http://dx.doi.org/10.1016/j.cub.2017.01.025

SUMMARY

Nodes of Ranvier in the axons of myelinated neurons are exemplars of the specialized cell surface domains typical of polarized cells. They are rich in voltage-gated sodium channels (Nav) and thus underpin rapid nerve impulse conduction in the vertebrate nervous system [1]. Although nodal proteins cluster in response to myelination, how myelin-forming glia influence nodal assembly is poorly understood. An axoglial adhesion complex comprising glial Neurofascin155 and axonal Caspr/Contactin flanks mature nodes [2]. We have shown that assembly of this adhesion complex at the extremities of migrating oligodendroglial processes promotes process convergence along the axon during central nervous system (CNS) node assembly [3]. Here we show that anchorage of this axoglial complex to the axon cytoskeleton is essential for efficient CNS node formation. When anchorage is disrupted, both the adaptor Protein 4.1B and the cytoskeleton protein βII spectrin are mislocalized in the axon, and assembly of the node of Ranvier is significantly delayed. Nodal proteins and migrating oligodendroglial processes are no longer juxtaposed, and single detached nodal complexes replace the symmetrical heminodes found in both the CNS and peripheral nervous system (PNS) during development. We propose that axoglial adhesion complexes contribute to the formation of an interface between cytoskeletal elements enriched in Protein 4.1B and βII spectrin and those enriched in nodal ankyrinG and βIV spectrin. This clusters nascent nodal complexes at heminodes and promotes their timely coalescence to form the mature node of Ranvier. These data demonstrate a role for the axon cytoskeleton in the assembly of a critical neuronal domain, the node of Ranvier.

RESULTS AND DISCUSSION

Voltage-gated sodium channels (Nav) clustered at nodes of Ranvier promote rapid nerve conduction in the myelinated nerves of vertebrates [1]. Central nervous system (CNS) nodes assemble in response to ensheathment by oligodendroglia when contiguous glial processes converge. The nodal gap is flanked by an intercellular adhesion complex comprising the glial isoform of Neurofascin (Nfasc155) with the axonal proteins Caspr and Contactin [2–8]; this complex also promotes the convergence of glial processes [3]. Oligodendroglia have an intrinsic ability to extend processes [9, 10], but whether the axon has a dynamic role in CNS node formation is unknown.

Extracellular Axoglial Adhesion Complexes Form When Caspr Is Uncoupled from Axonal Protein 4.1B

The C terminus of Caspr interacts with the axonal cytoskeleton adaptor protein Protein 4.1B [7, 11–16]. We asked whether this interaction influenced the migration of oligodendroglial processes by generating transgenic mice expressing either Caspr-GFP or Caspr lacking the 74 amino acids of its cytoplasmic C terminus, ΔC-Caspr-GFP, in Caspr$^{-/-}$ mice [17]. GFP fused to the C terminus of the transgenic proteins allowed confirmation of correct targeting. The antibody against wild-type (WT) Caspr recognizes ΔC-Caspr-GFP poorly (Figure 1A). Hence, anti-Caspr antibodies were used to detect WT Caspr and anti-GFP antibodies were used to detect transgenic Caspr for both western blotting and immunofluorescence. All immunofluorescence microscopy on CNS tissue was on teased fibers from the ventral funiculus of the spinal cord [3].

Caspr and ΔC-Caspr transgenic proteins were expressed at comparable levels in CNS axons (Figure 1A), and both formed extracellular ternary complexes visualized as electron-dense septate junctions between the base of the paranodal loops of myelin and the axolemma [2, 4, 5] (Figure 1B). Septate junctions are absent in Caspr null mice (Figure 1B) [4]. As found previously in the peripheral nervous system (PNS) [14], both Caspr and ΔC-Caspr transgenic proteins were targeted correctly in the absence of endogenous Caspr, since they colocalized with Claudin 11, a marker of oligodendroglial paranodal loops [3, 18], and Nfasc155, the glial component of the axoglial ternary complex (Figure 1C). At post-natal day 6 (P6), the convergence of myelinating processes in *ΔC-Caspr-GFP/Caspr*$^{-/-}$ mice appeared incomplete (Figure 1C), and this is addressed in more detail below.

When the extracellular axoglial adhesion complex is disturbed, mice perform poorly in tests of motor coordination [4, 19]. However, in the grid walking test [20], 5-week-old WT,

 © 2017 The Author(s). Published by Elsevier Ltd.
This is an open access article under the CC BY license (http://creativecommons.org/licenses/by/4.0/).

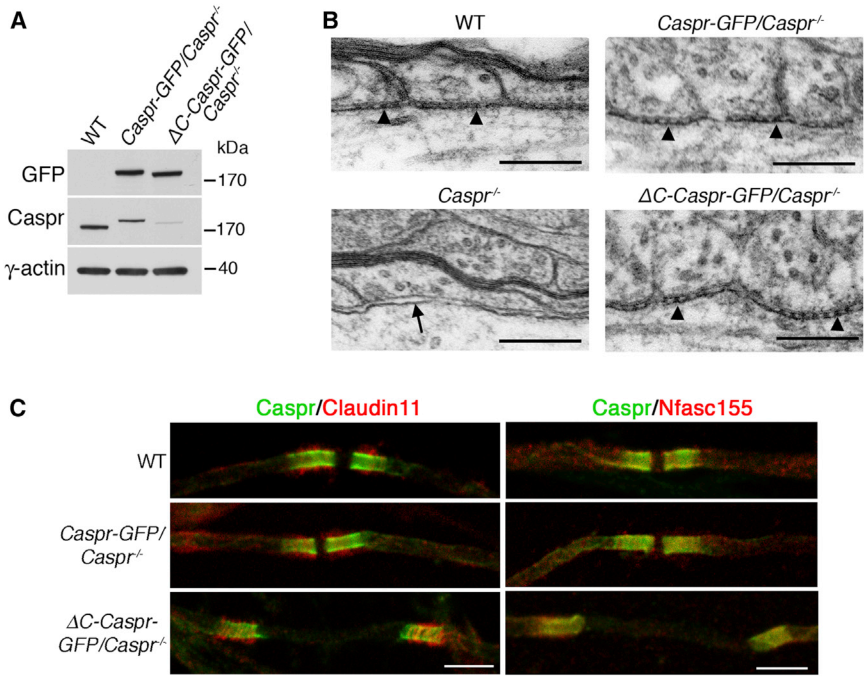

Figure 1. Extracellular Axoglial Adhesion in Caspr Null Mice Expressing Caspr without Its C Terminus

(A) Western blotting of spinal cord lysates on a 4%–12% NuPAGE gel showed that expression levels of Caspr-GFP and ΔC-Caspr-GFP were similar in Caspr null mice. Samples (20 μg) were blotted sequentially with mouse anti-GFP followed by rabbit anti-Caspr, with γ-actin blotted as a loading control in each lane. As expected, Caspr-GFP appears larger than WT Caspr and ΔC-Caspr-GFP is smaller than Caspr-GFP.

(B) Electron microscopy of paranodes from the ventral funiculus of the spinal cord of WT, *Caspr-GFP/Caspr*$^{-/-}$, and *ΔC-Caspr-GFP/Caspr*$^{-/-}$ mice at P41 showed the extracellular adhesion complex at the axoglial interface as electron-dense septate junctions. In *Caspr*$^{-/-}$ mice septate junctions were absent. The scale bars represent 1 μm.

(C) Immunofluorescence at P6 showed that Caspr, Caspr-GFP, and ΔC-Caspr-GFP colocalized with both the oligodendroglial paranodal loop marker Claudin 11 and the adhesion complex protein Nfasc155. The scale bars represent 5 μm.

Caspr-GFP/Caspr$^{-/-}$, and *ΔC-Caspr-GFP/Caspr*$^{-/-}$ mice were indistinguishable by the number of their foot faults; in contrast, *Caspr*$^{-/-}$ mice with disrupted axoglial adhesion complexes performed poorly ($p \leq 0.0005$) (WT, 7.4 ± 2.2; *Caspr-GFP/Caspr*$^{-/-}$, 7.7 ± 1.4; *ΔC-Caspr-GFP/Caspr*$^{-/-}$, 9.8 ± 1.7; *Caspr*$^{-/-}$, 38.8 ± 3.5; n = 3, mean ± SEM, Tukey's multiple comparison test).

The behavioral deficit in *Caspr*$^{-/-}$ mice was not caused by differences in myelin thickness, since there were no significant differences in g-ratio among the four genotypes at 5 weeks (WT, 0.75 ± 0.01; *Caspr-GFP/Caspr*$^{-/-}$, 0.74 ± 0.01; *ΔC-Caspr-GFP/Caspr*$^{-/-}$, 0.74 ± 0.01; *Caspr*$^{-/-}$, 0.74 ± 0.01; n = 3, mean ± SEM, Tukey's multiple comparison test). With no differences in myelin thickness, there were unlikely to be any differences in the number of paranodal loops among the four genotypes (Figure 1B). The number of oligodendroglia was also not significantly different in all four genotypes at 5 weeks (WT, 328 ± 16; *Caspr*$^{-/-}$, 296 ± 15; *Caspr-GFP/Caspr*$^{-/-}$, 355 ± 18; *ΔC-Caspr-GFP/Caspr*$^{-/-}$, 319 ± 13; n = 3, mean ± SEM, Tukey's multiple comparison test), showing that oligodendroglial differentiation was also unaffected.

Taken together, these data indicate that the extracellular axoglial adhesive complex was intact in *Caspr-GFP/Caspr*$^{-/-}$ and *ΔC-Caspr-GFP/Caspr*$^{-/-}$ mice, when compared to WT.

Anchorage of the Axoglial Adhesion Complex to Protein 4.1B Is Required for Heminode Formation

The convergence of symmetrical heminodes has been proposed to increase the efficiency of nodal assembly in the PNS [21–25]. We found that CNS heminodes were also evident, suggesting that these nascent clusters are a general feature of node formation (Figure 2A). However, when the axoglial adhesion complex was uncoupled from Protein 4.1B, either after complete loss of Caspr or when Caspr lacked its C terminus, symmetrical heminodes were no longer formed and a single nodal complex was always observed between converging processes (Figures 2B and 2C). Quantitation of ≥21 pairs of converging oligodendroglial processes per animal showed that the percentages of symmetrical heminodes were as follows: WT, 91 ± 2; *Caspr*$^{-/-}$, 0; *Caspr-GFP/Caspr*$^{-/-}$, 97 ± 2; and *ΔC-Caspr-GFP/Caspr*$^{-/-}$, 0 (n = 3, mean ± SEM). Note that ankyrinG was also associated with the axoglial junction (Figure 2C), where it interacts with Nfasc155 in oligodendroglia [26]. However, this interaction is not required for node assembly [3].

Our conclusion that heminode formation requires the linkage of Caspr to Protein 4.1B was supported by the fact that Protein 4.1B null mice also exhibited aberrant localization of nodal proteins, and symmetrical pairs of heminodes were never observed (Figure 2D).

Loss of Anchorage of the Axoglial Complex to Protein 4.1B Causes Mislocalization of Axonal Protein 4.1B and βII Spectrin

At P6 Protein 4.1B was depleted from the domains enriched in the axoglial complex in *ΔC-Caspr-GFP/Caspr*$^{-/-}$ compared to *Caspr-GFP/Caspr*$^{-/-}$ mice (Figure 3, arrowheads). Perhaps even more strikingly, Protein 4.1B was mislocalized in *ΔC-Caspr-GFP/Caspr*$^{-/-}$ to the regions of the axon between converging processes (Figure 3, asterisk). Protein 4.1B is believed to be linked to the axonal cytoskeletal proteins αII spectrin and βII spectrin at the paranodes in the PNS and CNS [27]. In support of this, βII spectrin followed the mislocalization of Protein 4.1B (Figure 3, asterisk), although it was not obviously depleted at the axoglial junction. Mislocalization of both Protein 4.1B and βII spectrin was always observed in *ΔC-Caspr-GFP/Caspr*$^{-/-}$ mice but was never observed in *Caspr-GFP/Caspr*$^{-/-}$ mice.

The axon initial segment (AIS) is the axon's proximal domain and is enriched in proteins also found at nodes of Ranvier,

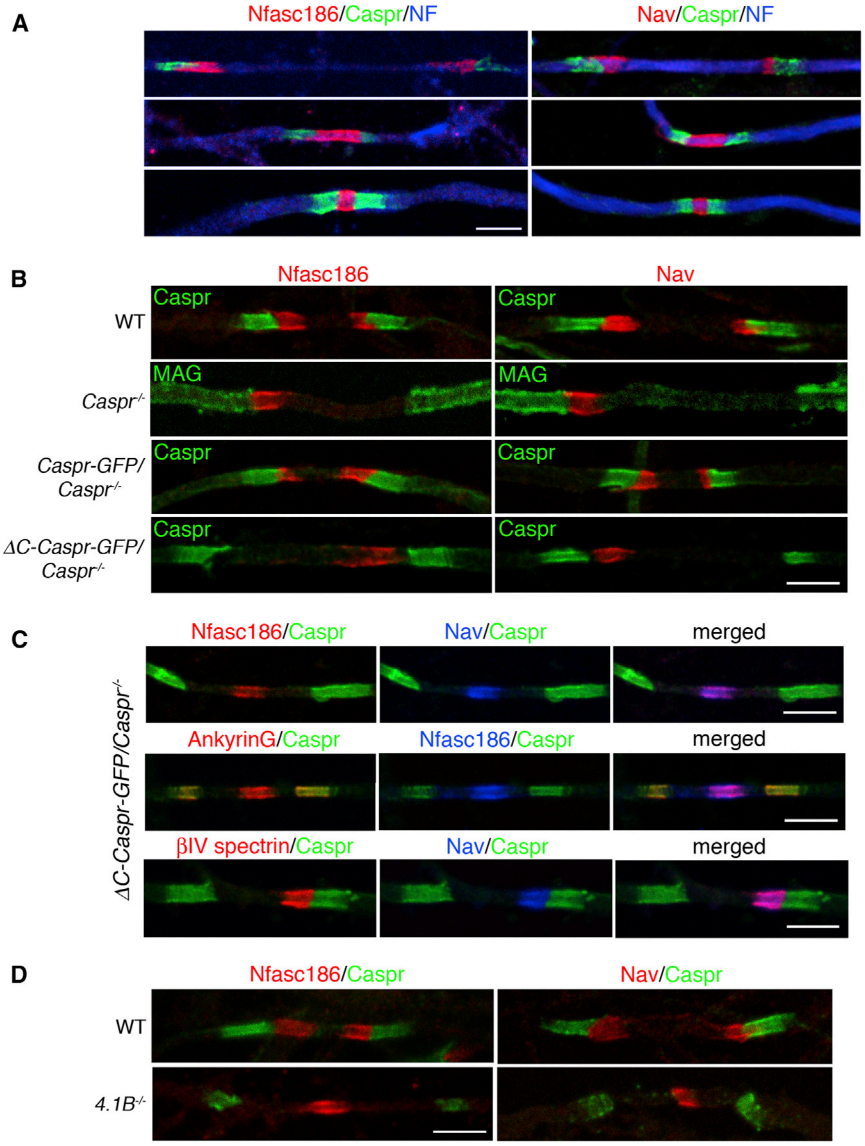

Figure 2. Mislocalization of Nodal Complexes in Caspr Null and *ΔC-Caspr-GFP/Caspr$^{-/-}$* Mice

(A) Although most nodes are mature in WT nerves at P6 (see Figure 4), earlier stages of heminode fusion can be detected by immunofluorescence for either Nfasc186 or Nav (both nodal proteins) with Caspr and Neurofilament (NF).

(B) Immunofluorescence at P6 showed that complete disruption of the axoglial complex (*Caspr$^{-/-}$*) or removal of the Protein 4.1B binding site in Caspr (*ΔC-Caspr-GFP/Caspr$^{-/-}$*) abolished symmetrical heminode formation and resulted in a single nodal complex. Heminode formation was rescued with transgenic Caspr (*Caspr-GFP/Caspr$^{-/-}$*).

(C) Immunostaining at P6 showed that the mislocalized nodal complex in *ΔC-Caspr-GFP/Caspr$^{-/-}$* mice contained Nav, Nfasc186, βIV spectrin, and ankyrinG.

(D) Immunofluorescence at P6 for Nfasc186 and Nav in the absence of Protein 4.1B (*4.1B$^{-/-}$*) showed that the nodal complex was mislocalized.

The scale bars represent 5 μm. See also Figure S1.

including Nav, Nfasc186, βIV spectrin, and ankyrinG. It has been argued that βII spectrin and its associated axonal cytoskeletal proteins contribute to an intra-axonal boundary that excludes βIV spectrin- and ankyrinG-associated proteins, such as Nfasc186, and defines the extent of the AIS [28]. It has also been proposed that the βII spectrin-based cytoskeleton forms a boundary at the paranode, since loss of βII spectrin leads to mislocalization of adjacent juxtaparanodal proteins even when the extracellular axoglial junction is intact [29]. Our observations are consistent with this model. When the axoglial complex was uncoupled from the axonal cytoskeleton, Protein 4.1B and βII spectrin were no longer contained by the tips of myelinating processes (Figure 2C). Nevertheless, it appeared that the intra-axonal interface remained intact but mislocalized, since βII spectrin was still largely excluded from the nodal complex (Figure 3). This was probably because ankyrinG and βIV spectrin remained associated with the singular nodal complex (Figure 3) and linked to the underlying actin cytoskeleton [30].

Disruption of the Axoglial Link to the Axon Cytoskeleton Delays Node Formation

We have found previously that disrupting axoglial adhesion in the CNS by eliminating glial Nfasc155 results in a marked delay in process migration and a consequent increase in the gap between converging oligodendroglial processes [3]. This delay in forming the mature node is also seen with the loss of Caspr (Figures 4A and 4B). However, Caspr lacking its cytoplasmic C terminus also delayed the migration of myelinating processes to the same degree as complete loss of Caspr (Figures 4A and 4B). As in Neurofascin mutants, nodes with normal widths were eventually formed (Figure 4B) [3]. The absence of Protein 4.1B was earlier shown to result in the same heminodal phenotype seen in *ΔC-Caspr-GFP/Caspr$^{-/-}$* mice (Figure 2D), and delayed migration was recapitulated in Protein 4.1B null mice (Figures 4C and 4D). We have previously shown that nodal Neurofascins are capable of assembling nodes in the complete absence of paranodal axoglial junctions, so these structures are not essential for node assembly in the CNS, but they clearly make it much more efficient [3, 19].

Conclusions

Efficient convergence of myelinating processes to form the CNS node of Ranvier is promoted by an axoglial adhesion complex that must also be anchored to the axonal cytoskeleton via the adaptor Protein 4.1B. Loss of this interaction causes mislocalization of Protein 4.1B and βII spectrin, and

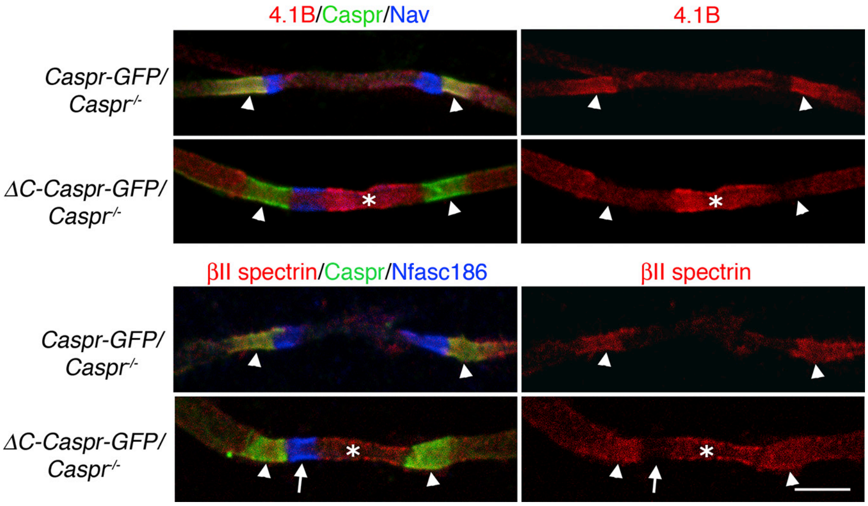

Figure 3. Mislocalization of Axonal Protein 4.1B and βII Spectrin in *ΔC-Caspr-GFP/Caspr$^{-/-}$* Mice

Immunostaining at P6 showed depletion of Protein 4.1B at the axoglial junction in *ΔC-Caspr-GFP/Caspr$^{-/-}$* mice (arrowheads) and concomitant invasion of the protein into the axon between converging processes (asterisk). Although βII spectrin persisted at the axoglial junction (arrowheads) in *ΔC-Caspr-GFP/Caspr$^{-/-}$* mice, it was also mislocalized between converging preocesses (asterisk). Nevertheless, it was still largely excluded from the nodal complex (arrow), as observed in control *Caspr-GFP/Caspr$^{-/-}$* mice. The scale bar represents 5 μm.

nascent clusters of nodal proteins at heminodes are no longer formed at the leading edge of converging myelinating processes. Hence, symmetrical heminodes no longer fuse to form mature nodes, and there is a significant delay in the convergence of myelinating processes. We propose that, as oligodendroglial processes converge, axoglial adhesion complexes define an interface between axonal cytoskeletal elements enriched in Protein 4.1B and βII spectrin and those enriched in ankyrinG and βIV spectrin that associate with nodal proteins. One possibility is that βII spectrin and βIV spectrin compete for interaction with the actin cytoskeleton. Our current work supports earlier proposals that the axon cytoskeleton has a general role in organizing and defining specialized domains in axons [28, 29]. Furthermore, this report extends our understanding of the intra-axonal boundary by showing that Protein 4.1B is an essential axonal component recruited and localized by neuron-glia interactions.

EXPERIMENTAL PROCEDURES

Generation of Mutant Mice

All animal work conformed to UK legislation (Scientific Procedures) Act 1986 and to the University of Edinburgh Ethical Review policy. Generation of cDNAs encoding full-length rat Caspr and Caspr lacking its intracellular domain, both fused at their C termini to GFP was as previously described [17]. Both constructs were released from the pEGFPN1 vector with EcoR1 and Not1, blunted, and cloned into a blunted Xho1 site in the Thy1 promoter vector pTSC21k [31]. The transgenes were released by digestion with Not1 and transgenic mice were generated as described [32]. Founders were identified and backcrossed to the C57BL/6 background for at least six generations before data collection. Transgenic mice were interbred with Caspr$^{+/-}$ mice, also on

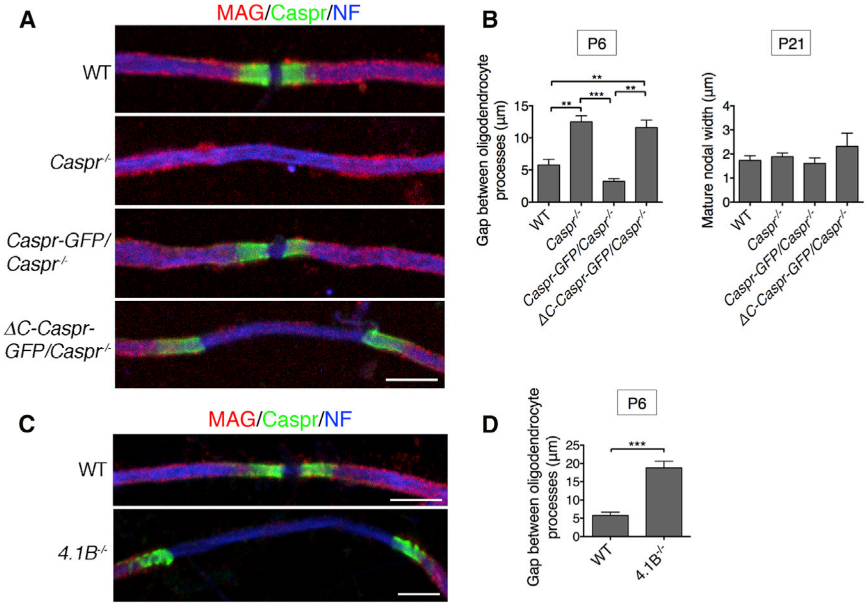

Figure 4. Interaction of the Intracellular Domain of Caspr with Axonal Protein 4.1B Promotes Oligodendroglial Process Migration

(A) Immunofluorescence at P6 for Myelin-Associated Glycoprotein (MAG), the axonal marker Neurofilament-H (NF), Caspr-GFP, and ΔC-Caspr-GFP suggested that the migration of oligodendroyte processes was delayed in *Caspr$^{-/-}$* and *ΔC-Caspr-GFP/Caspr$^{-/-}$* mice.

(B) Quantitation of the gaps between the tips of migrating processes at P6 and P21. Caspr-GFP rescued the delay in process migration observed in *Caspr$^{-/-}$* mice at P6 whereas ΔC-Caspr-GFP did not. However, by P21 there was no significant difference in the width of the nodal gap among the four genotypes. Values are means ± SEM (n = 3 mice per genotype, a minimum of 50 gaps between converging pairs of processes were measured per mouse; ***p ≤ 0.001 and **p ≤ 0.01, Tukey's multiple comparison test).

(C and D) Immunofluorescence (C) and quantitative analysis (D) of Protein 4.1B null (*4.1B$^{-/-}$*) spinal cord fibers at P6 (as in A and B) showed that the convergence of oligodendroglial processes was also significantly delayed compared with WT fibers. Values are means ± SEM (n = 3 mice per genotype, a minimum of 50 gaps between converging pairs of processes were measured per mouse; ***p ≤ 0.001, unpaired Student's t test).

The scale bars represent 5 μm.

a C57BL/6 background. Generation and characterization of Caspr null and Protein 4.1B null mice have been described [33, 34].

Antibodies and Microscopy

Immunostaining and western blotting of teased fiber preparations from the ventral funiculus of the cervical spinal cord and transverse cryosections of spinal cord were as described [3, 32]. Chicken antibodies versus GFP were from Abcam (1:1,000), mouse antibodies versus GFP were from Roche (1:2,000), mouse anti-APC/CC1 was from Oncogene (1:100), rabbit anti-Protein 4.1B was a gift from Dr. J.-A. Girault (1:500), and mouse anti-βII spectrin was from BD Biosciences (1:100). All other antibodies have been described [2, 32]. Samples were mounted in Vectashield (Vector Laboratories). Confocal microscopy was performed using a Leica TCL-SL microscope with a 1.4 numerical aperture (NA) 63× objective. Conventional fluorescence microscopy was with an Olympus microscope (BX60) with a 0.75 NA 40× objective lens, and images were captured using a camera (Orca-ER, Hamamatsu) and Improvision Openlab software. Electron microscopy of longitudinal sections of ventral funiculus was as described but using a JEOL JEM1400Plus electron microscope [35].

Morphometry and Behavioral Testing

The gaps between converging oligodendroglial processes were measured using Openlab software after image acquisition using conventional fluorescence microscopy. The total number of spinal cord oligodendroglia was quantitated in each transverse section, after immunostaining with anti-APC antibody, with ImageJ 1.47t (NIH) after montage construction (five spinal cord sections per animal, three animals per genotype minimum) [3]. Statistical analysis was performed with the GraphPad Prism 5.0c. The grid walking test was performed as described [20]. Mice were familiarized with the grid apparatus for 5 min of free walking. The following day, each animal was video recorded for 5 min while walking on the mesh, and the number of hindlimb mistakes was counted. G-ratios were measured as described [36].

SUPPLEMENTAL INFORMATION

Supplemental Information includes one figure and can be found with this article online at http://dx.doi.org/10.1016/j.cub.2017.01.025.

AUTHOR CONTRIBUTIONS

V.B. and P.J.B. designed the study. V.B. and D.L.S. performed the experiments. C.F.-S. and E.P. provided essential resources. V.B., D.L.S., and P.J.B. analyzed the data and wrote the paper.

ACKNOWLEDGMENTS

We thank Qiushi Li and Heather Anderson for excellent assistance. This work was supported by the Medical Research Council (MR/L011379/1) and the Wellcome Trust (107008/Z/15/Z).

Received: March 3, 2016
Revised: December 17, 2016
Accepted: January 12, 2017
Published: March 16, 2017

REFERENCES

1. Huxley, A.F., and Stämpfli, R. (1949). Evidence for saltatory conduction in peripheral myelinated nerve fibres. J. Physiol. *108*, 315–339.
2. Sherman, D.L., Tait, S., Melrose, S., Johnson, R., Zonta, B., Court, F.A., Macklin, W.B., Meek, S., Smith, A.J., Cottrell, D.F., and Brophy, P.J. (2005). Neurofascins are required to establish axonal domains for saltatory conduction. Neuron *48*, 737–742.
3. Zonta, B., Tait, S., Melrose, S., Anderson, H., Harroch, S., Higginson, J., Sherman, D.L., and Brophy, P.J. (2008). Glial and neuronal isoforms of Neurofascin have distinct roles in the assembly of nodes of Ranvier in the central nervous system. J. Cell Biol. *181*, 1169–1177.
4. Bhat, M.A., Rios, J.C., Lu, Y., Garcia-Fresco, G.P., Ching, W., St Martin, M., Li, J., Einheber, S., Chesler, M., Rosenbluth, J., et al. (2001). Axon-glia interactions and the domain organization of myelinated axons requires neurexin IV/Caspr/Paranodin. Neuron *30*, 369–383.
5. Boyle, M.E., Berglund, E.O., Murai, K.K., Weber, L., Peles, E., and Ranscht, B. (2001). Contactin orchestrates assembly of the septate-like junctions at the paranode in myelinated peripheral nerve. Neuron *30*, 385–397.
6. Charles, P., Tait, S., Faivre-Sarrailh, C., Barbin, G., Gunn-Moore, F., Denisenko-Nehrbass, N., Guennoc, A.M., Girault, J.A., Brophy, P.J., and Lubetzki, C. (2002). Neurofascin is a glial receptor for the paranodin/Caspr-contactin axonal complex at the axoglial junction. Curr. Biol. *12*, 217–220.
7. Menegoz, M., Gaspar, P., Le Bert, M., Galvez, T., Burgaya, F., Palfrey, C., Ezan, P., Arnos, F., and Girault, J.A. (1997). Paranodin, a glycoprotein of neuronal paranodal membranes. Neuron *19*, 319–331.
8. Peles, E., Nativ, M., Lustig, M., Grumet, M., Schilling, J., Martinez, R., Plowman, G.D., and Schlessinger, J. (1997). Identification of a novel contactin-associated transmembrane receptor with multiple domains implicated in protein-protein interactions. EMBO J. *16*, 978–988.
9. Bechler, M.E., Byrne, L., and Ffrench-Constant, C. (2015). CNS Myelin Sheath Lengths Are an Intrinsic Property of Oligodendrocytes. Curr. Biol. *25*, 2411–2416.
10. Lee, S., Leach, M.K., Redmond, S.A., Chong, S.Y., Mellon, S.H., Tuck, S.J., Feng, Z.Q., Corey, J.M., and Chan, J.R. (2012). A culture system to study oligodendrocyte myelination processes using engineered nanofibers. Nat. Methods *9*, 917–922.
11. Buttermore, E.D., Dupree, J.L., Cheng, J., An, X., Tessarollo, L., and Bhat, M.A. (2011). The cytoskeletal adaptor protein band 4.1B is required for the maintenance of paranodal axoglial septate junctions in myelinated axons. J. Neurosci. *31*, 8013–8024.
12. Einheber, S., Meng, X., Rubin, M., Lam, I., Mohandas, N., An, X., Shrager, P., Kissil, J., Maurel, P., and Salzer, J.L. (2013). The 4.1B cytoskeletal protein regulates the domain organization and sheath thickness of myelinated axons. Glia *61*, 240–253.
13. Gollan, L., Sabanay, H., Poliak, S., Berglund, E.O., Ranscht, B., and Peles, E. (2002). Retention of a cell adhesion complex at the paranodal junction requires the cytoplasmic region of Caspr. J. Cell Biol. *157*, 1247–1256.
14. Horresh, I., Bar, V., Kissil, J.L., and Peles, E. (2010). Organization of myelinated axons by Caspr and Caspr2 requires the cytoskeletal adapter protein 4.1B. J. Neurosci. *30*, 2480–2489.
15. Ohara, R., Yamakawa, H., Nakayama, M., and Ohara, O. (2000). Type II brain 4.1 (4.1B/KIAA0987), a member of the protein 4.1 family, is localized to neuronal paranodes. Brain Res. Mol. Brain Res. *85*, 41–52.
16. Denisenko-Nehrbass, N., Oguievetskaia, K., Goutebroze, L., Galvez, T., Yamakawa, H., Ohara, O., Carnaud, M., and Girault, J.A. (2003). Protein 4.1B associates with both Caspr/paranodin and Caspr2 at paranodes and juxtaparanodes of myelinated fibres. Eur. J. Neurosci. *17*, 411–416.
17. Bonnon, C., Goutebroze, L., Denisenko-Nehrbass, N., Girault, J.A., and Faivre-Sarrailh, C. (2003). The paranodal complex of F3/contactin and caspr/paranodin traffics to the cell surface via a non-conventional pathway. J. Biol. Chem. *278*, 48339–48347.
18. Gow, A., Southwood, C.M., Li, J.S., Pariali, M., Riordan, G.P., Brodie, S.E., Danias, J., Bronstein, J.M., Kachar, B., and Lazzarini, R.A. (1999). CNS myelin and sertoli cell tight junction strands are absent in Osp/claudin-11 null mice. Cell *99*, 649–659.
19. Zhang, A., Desmazieres, A., Zonta, B., Melrose, S., Campbell, G., Mahad, D., Li, Q., Sherman, D.L., Reynolds, R., and Brophy, P.J. (2015). Neurofascin 140 is an embryonic neuronal neurofascin isoform that promotes the assembly of the node of Ranvier. J. Neurosci. *35*, 2246–2254.
20. Chao, O.Y., Pum, M.E., Li, J.S., and Huston, J.P. (2012). The grid-walking test: assessment of sensorimotor deficits after moderate or severe dopamine depletion by 6-hydroxydopamine lesions in the dorsal striatum and medial forebrain bundle. Neuroscience *202*, 318–325.

21. Ching, W., Zanazzi, G., Levinson, S.R., and Salzer, J.L. (1999). Clustering of neuronal sodium channels requires contact with myelinating Schwann cells. J. Neurocytol. *28*, 295–301.
22. Dugandzija-Novaković, S., Koszowski, A.G., Levinson, S.R., and Shrager, P. (1995). Clustering of Na+ channels and node of Ranvier formation in remyelinating axons. J. Neurosci. *15*, 492–503.
23. Feinberg, K., Eshed-Eisenbach, Y., Frechter, S., Amor, V., Salomon, D., Sabanay, H., Dupree, J.L., Grumet, M., Brophy, P.J., Shrager, P., and Peles, E. (2010). A glial signal consisting of gliomedin and NrCAM clusters axonal Na+ channels during the formation of nodes of Ranvier. Neuron *65*, 490–502.
24. Schafer, D.P., Custer, A.W., Shrager, P., and Rasband, M.N. (2006). Early events in node of Ranvier formation during myelination and remyelination in the PNS. Neuron Glia Biol. *2*, 69–79.
25. Vabnick, I., Novaković, S.D., Levinson, S.R., Schachner, M., and Shrager, P. (1996). The clustering of axonal sodium channels during development of the peripheral nervous system. J. Neurosci. *16*, 4914–4922.
26. Chang, K.J., Zollinger, D.R., Susuki, K., Sherman, D.L., Makara, M.A., Brophy, P.J., Cooper, E.C., Bennett, V., Mohler, P.J., and Rasband, M.N. (2014). Glial ankyrins facilitate paranodal axoglial junction assembly. Nat. Neurosci. *17*, 1673–1681.
27. Ogawa, Y., Schafer, D.P., Horresh, I., Bar, V., Hales, K., Yang, Y., Susuki, K., Peles, E., Stankewich, M.C., and Rasband, M.N. (2006). Spectrins and ankyrinB constitute a specialized paranodal cytoskeleton. J. Neurosci. *26*, 5230–5239.
28. Galiano, M.R., Jha, S., Ho, T.S., Zhang, C., Ogawa, Y., Chang, K.J., Stankewich, M.C., Mohler, P.J., and Rasband, M.N. (2012). A distal axonal cytoskeleton forms an intra-axonal boundary that controls axon initial segment assembly. Cell *149*, 1125–1139.
29. Zhang, C., Susuki, K., Zollinger, D.R., Dupree, J.L., and Rasband, M.N. (2013). Membrane domain organization of myelinated axons requires βII spectrin. J. Cell Biol. *203*, 437–443.
30. Bennett, V., and Baines, A.J. (2001). Spectrin and ankyrin-based pathways: metazoan inventions for integrating cells into tissues. Physiol. Rev. *81*, 1353–1392.
31. Lüthi, A., Van der Putten, H., Botteri, F.M., Mansuy, I.M., Meins, M., Frey, U., Sansig, G., Portet, C., Schmutz, M., Schröder, M., et al. (1997). Endogenous serine protease inhibitor modulates epileptic activity and hippocampal long-term potentiation. J. Neurosci. *17*, 4688–4699.
32. Zonta, B., Desmazieres, A., Rinaldi, A., Tait, S., Sherman, D.L., Nolan, M.F., and Brophy, P.J. (2011). A critical role for Neurofascin in regulating action potential initiation through maintenance of the axon initial segment. Neuron *69*, 945–956.
33. Cifuentes-Diaz, C., Chareyre, F., Garcia, M., Devaux, J., Carnaud, M., Levasseur, G., Niwa-Kawakita, M., Harroch, S., Girault, J.A., Giovannini, M., and Goutebroze, L. (2011). Protein 4.1B contributes to the organization of peripheral myelinated axons. PLoS ONE *6*, e25043.
34. Gollan, L., Salomon, D., Salzer, J.L., and Peles, E. (2003). Caspr regulates the processing of contactin and inhibits its binding to neurofascin. J. Cell Biol. *163*, 1213–1218.
35. Gillespie, C.S., Sherman, D.L., Fleetwood-Walker, S.M., Cottrell, D.F., Tait, S., Garry, E.M., Wallace, V.C., Ure, J., Griffiths, I.R., Smith, A., and Brophy, P.J. (2000). Peripheral demyelination and neuropathic pain behavior in periaxin-deficient mice. Neuron *26*, 523–531.
36. Sherman, D.L., Krols, M., Wu, L.M., Grove, M., Nave, K.A., Gangloff, Y.G., and Brophy, P.J. (2012). Arrest of myelination and reduced axon growth when Schwann cells lack mTOR. J. Neurosci. *32*, 1817–1825.

Current Biology, Volume *27*

Supplemental Information

Assembly of CNS Nodes of Ranvier in Myelinated Nerves Is Promoted by the Axon Cytoskeleton

Veronica Brivio, Catherine Faivre-Sarrailh, Elior Peles, Diane L. Sherman, and Peter J. Brophy

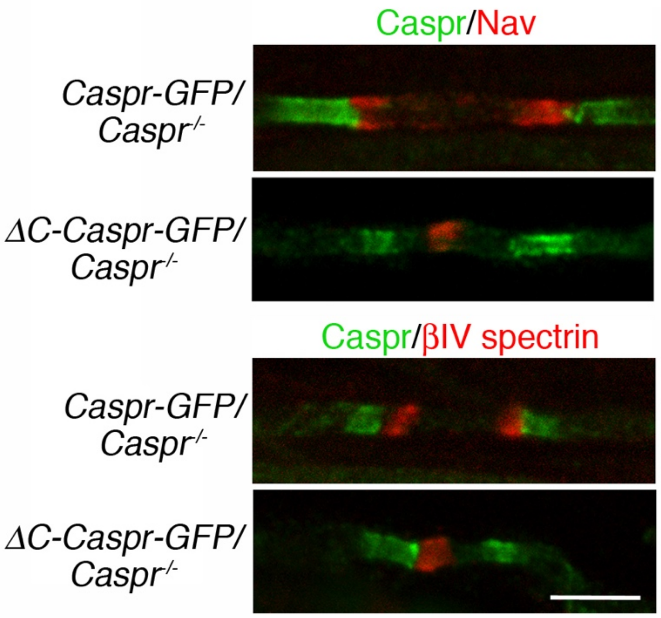

Figure S1. The absence of the Protein 4.1B binding domain of Caspr also impairs the formation of heminodal clusters in the PNS. Related to Figure 2.

Immunofluorescence analysis at P3 of sciatic nerves from *Caspr-GFP/Caspr*$^{-/-}$ and *ΔC-Caspr-GFP/Caspr*$^{-/-}$ mice for Caspr and the nodal proteins Nav and βIV spectrin. Quantitation of ≥ 63 pairs of converging Schwann cells for the nodal proteins Nav, βIV spectrin or ankyrinG for each genotype showed that the percentages of symmetrical heminodes were as follows *Caspr-GFP/Caspr*$^{-/-}$, 89.0 ± 0.3 (n = 2); *C-Caspr-GFP/Caspr*$^{-/-}$, 12.0 ± 3.0 (n = 3); mean ± SEM, $P \leq 0.0002$, Unpaired Student's t test. The scale bar represents 5 µm.
